# Modulation of Intestinal ILC3 for the Treatment of Type 1 Diabetes

**DOI:** 10.3389/fimmu.2021.653560

**Published:** 2021-06-03

**Authors:** Ivana Stojanović, Tamara Saksida, Đorđe Miljković, Nada Pejnović

**Affiliations:** Department of Immunology, Institute for Biological Research “Siniša Stanković” – National Institute of the Republic of Serbia, University of Belgrade, Belgrade, Serbia

**Keywords:** type 3 innate lymphoid cells (ILC3), type 1 diabetes (T1D), gut-associated lymphoid tissue (GALT), regulatory T cells (Treg), interleukin-22 (IL-22), interleukin-2 (IL-2)

## Abstract

Gut-associated lymphoid tissue (GALT) is crucial for the maintenance of the intestinal homeostasis, but it is also the potential site of the activation of autoreactive cells and initiation/propagation of autoimmune diseases in the gut and in the distant organs. Type 3 innate lymphoid cells (ILC3) residing in the GALT integrate signals from food ingredients and gut microbiota metabolites in order to control local immunoreactivity. Notably, ILC3 secrete IL-17 and GM-CSF that activate immune cells in combating potentially pathogenic microorganisms. ILC3 also produce IL-22 that potentiates the strength and integrity of epithelial tight junctions, production of mucus and antimicrobial peptides thus enabling the proper function of the intestinal barrier. The newly discovered function of small intestine ILC3 is the secretion of IL-2 and the promotion of regulatory T cell (Treg) generation and function. Since the intestinal barrier dysfunction, together with the reduction in small intestine ILC3 and Treg numbers are associated with the pathogenesis of type 1 diabetes (T1D), the focus of this article is intestinal ILC3 modulation for the therapy of T1D. Of particular interest is free fatty acids receptor 2 (FFAR2), predominantly expressed on intestinal ILC3, that can be stimulated by available selective synthetic agonists. Thus, we propose that FFAR2-based interventions by boosting ILC3 beneficial functions may attenuate autoimmune response against pancreatic β cells during T1D. Also, it is our opinion that treatments based on ILC3 stimulation by functional foods can be used as prophylaxis in individuals that are genetically predisposed to develop T1D.

## Introduction

Type 1 diabetes (T1D) is an autoimmune disease that is characterized by low insulin concentration and hyperglycemia. The autoimmune process in pancreatic islets can last for years before the clinical signs of the disease appear. This process is initiated by autoreactive effector T cells including CD4^+^ and CD8^+^ cells and it is characterized by high levels of proinflammatory cytokines IL-1β, TNF and IFN-γ (1). The described events are accompanied by decreased numbers and/or defective function of regulatory T cells (Treg) that have an immunosuppressive role and maintain immune tolerance by producing IL-10 and TGF-β and by other mechanisms (2). The overall outcome is the destruction of pancreatic β cells that leads to reduced or completely absent insulin production (1, 3).

Many environmental factors including food ingredients (β-casein or bovine insulin from cow’s milk, gluten), exposure to infectious agents (enteroviruses), and intestinal microbiota dysbiosis (due to antibiotics, alcohol abuse, inadequate diet or chronic diseases) are believed to be the reason for the dramatic increase in T1D incidence in people under the age of 18, but also in older adults (4–6).

It is becoming increasingly clear that T1D pathogenesis is linked to the complex interaction between the gut-associated lymphoid tissue (GALT) and the gut microbiota (7). Intestinal barrier serves as an integrator of signals coming from the gut lumen and it is comprised of mucus layer leaning on tightly connected epithelial cells (physical border) and mediators secreted by epithelial cells and immune cells (functional border). GALT cells maintain immune tolerance to food constituents and commensal microbes. The reduction or improper function of GALT-residing tolerogenic dendritic cells (DC) and Treg enables the impairment of oral tolerance (8–10), that may lead to T1D initiation mediated by autoreactive T cells present in the intestinal lamina propria (11, 12). In such case, antigens sampled from the gut might activate β cell-reactive immune cells directly *via* molecular mimicry or indirectly by the bystander activation during the immune response towards gut microorganisms (13). The close link between the gut and the pancreas is exemplified in the finding that pancreatic lymph nodes can drain antigens from the duodenum that leads to Treg induction in GALT and development of oral tolerance (14). Therefore, maintaining a balance between effector T cells and Treg in the gut and pancreatic lymph nodes is essential for sustaining tolerance to islet antigens and prevention of autoreactive T lymphocytes activation and migration to the pancreas where they can initiate β-cell destruction.

## Gut-Pancreas Axis

The impaired function of the intestinal barrier and dysbiosis precede the development of T1D both in humans and mice. The loss of gut barrier integrity and low-grade intestinal inflammation were discovered in first-degree relatives of T1D patients that are at high-risk of disease development (15–17). The same was confirmed in new-onset and long-term T1D patients (17, 18). The altered microbiota content in T1D patients were found in many studies worldwide as reviewed by Marietta et al. (19).

Increased intestinal permeability and the lack of oral tolerance to ovalbumin was found in 4-6 weeks old, insulitis-free nonobese diabetic (NOD) mice that spontaneously develop T1D (10). Also, these mice had diminished mucus production, lower levels of secretory IgA and increased Th17 and type 3 innate lymphoid cells (ILC3) numbers in the small intestine lamina propria. This coincided with the significant reduction of tolerogenic DC and Treg in the gut-draining lymph nodes during prediabetic stage (10).

There are very few studies that address the activation of autoreactive cells in GALT and their causal link to pancreas autoimmunity. Our recent study implies that activation of insulin-specific CD4^+^ T cells can occur in the GALT as these cells are present in Peyer’s patches of prediabetic NOD and healthy C57BL/6 mice (11). Also, a study that used a β cell–specific TCR-transgenic mouse model has shown that islet-specific T cells activated in the intestinal lamina propria migrated to the pancreatic lymph nodes and the islets causing autoimmune diabetes (20). Further, it was demonstrated that the infection with *Fusobacteria* activates β cell-reactive CD8^+^ T cells by molecular mimicry within GALT of transgenic NOD mice (12). In addition to the possibility of autoreactive cell activation in the GALT, it was shown that gut microbiota can migrate to the pancreatic lymph nodes where it acts through NOD2 receptors to accelerate the onset of streptozotocin-induced T1D in mice (21). Human studies about the autoreactive cells activation within the GALT indirectly suggest that ingested food or bacterial antigens stimulate the production of β cell-specific autoantibodies *via* molecular mimicry. Examples can be found in reports of Auricchio et al. (22) and Niegowska et al. (23) where data about crossreactivity between β cell antigens and antigens derived from gluten or *Mycobacterium avium* subspecies *paratuberculosis*, a bacterium found in cow’s milk, were suggested. Also, higher density of intraepithelial CD3^+^ and γδ cells and activated CD25^+^ in lamina propria and lower numbers of FoxP3^+^ cells in the jejunal mucosa of T1D patients were found (22, 24, 25). In general, individuals with T1D exhibit increased markers of inflammation within GALT suggesting its association with disease development (26).

Prevention or treatment of human T1D through diet-based interventions proved to be very difficult (27). However, a forced change in microbiota content through fecal microbiota transplantation from healthy donors to early-onset T1D patients successfully halted a decline in endogenous insulin production and down-regulated colonic CD4^+^ cell count, thus further confirming the importance of microbiota content for T1D control (28). In contrast to scarce data in humans, numerous studies provide evidence about prevention or treatment of animal T1D through diet or modulation of microbiota (29, 30). To mention a few: NOD mice fed with a fiber-rich diet had decreased T1D incidence and lower proportion of autoantigen-specific CD8^+^ lymphocytes in the spleen (31), supplementation with bacterial metabolite butyrate decreased severity of insulitis in NOD mice and their offspring by promoting Treg proliferation in GALT and their migration to the pancreas (32, 33), administration of probiotics exerted beneficial effects in T1D in mice (34–36).

The majority of available data point to the importance of intestinal Treg and their suppressive properties in the prevention and/or treatment of T1D (8, 9). ILC3 have recently been identified as cells critical for maintenance and regulation of mucosal homeostasis in mice and humans (37), but their role in the initiation or development of T1D is largely unknown. This Perspective review will specifically discuss ILC3 biology and their hypothetical role in pancreatic autoimmunity along with possibilities of ILC3-targeted therapies.

## Intestinal ILC3

Immature ILC develop in bone marrow from common lymphoid progenitor and they generally migrate to mucosal tissues, but can also be found in other lymphoid tissues such as spleen and lymph nodes and non-lymphoid organs skin, liver, brain and pancreas (38–41). As reviewed by Guia et al. (42), ILC3 differentiation process is similar in humans and mice. ILC3 can be identified as the innate counterpart of Th17 cells due to their mandatory expression of retinoid-related orphan receptor γt (RORγt). ILC3 exist in at least two subsets that differ developmentally, transcriptionally and functionally: lymphoid tissue inducer cells (LTi)-like ILC3 (characterized by surface expression of CCR6) and natural cytotoxicity receptor (NCR)^+^ ILC3 that express NKp46 in mice (43) and NKp44 in humans (44). However, human ILC3 can also express NKp46 and their distribution in skin and intestine was found very similar in humans and mice (45). ILC3 are generally sedentary (46, 47), although in some human pathological conditions differentiated ILC3 were found in the bloodstream (48). Therefore, their regular divisions driven by different internal and environmental signals is essential for their maintenance in the tissues. ILC3 proliferation is stimulated by cytokines, such as IL-18 in human tonsils (49), or combination of tumor necrosis factor-like cytokine 1A, IL-1β, IL-23 and IL-2 in both human and mouse intestinal tissue (50, 51). The major environmental stimuli for murine intestinal ILC3 proliferation are short chain free fatty acids (SCFA) and vitamins A and D (52, 53).

Mature ILC3 develop in the lamina propria of the intestine due to specific differentiation factors (retinoic acid, polyphenols and microbiota) (37). Mouse studies indicate that intestinal ILC3 express integrin α4β7. Their specific signature is the expression of GPR183, a receptor for oxysterols that recruits ILC3 to the small intestine and regulates their migration to the cryptopatches and positioning in the mesenteric lymph nodes. The expression of GPR109A (a receptor for butyrate) dictates ILC3 distribution in Peyer’s patches, while distinct pattern of chemokine receptors drives their migration to the specific sites in the GALT such as mesenteric lymph nodes (CCR7), microvilli (CXCR6) or lamina propria (CCR9) (reviewed in 54). In addition, intestinal ILC3 exhibit high free fatty acid receptor (FFAR) expression in contrast to spleen ILC3, for example (55).

Intestinal human and mouse ILC3 are critical for the generation of the organized lymphoid tissue in the intestinal wall during development (LTi-like cells) and they regulate microbiota content and the integrity of the intestinal barrier (46, 56). Mouse ILC3 sense environmental cues either coming from the food or microbiota metabolism products by expressing numerous receptors: retinoic acid receptor (RAR) (57), vitamin D receptor (VDR) (58), aryl hydrocarbon receptor (AhR) (56, 59), or FFAR (55). Also, gut ILC3 respond to cytokines predominantly produced by myeloid cells (IL-1β, IL-23, IL-18 and TNF). In response to these triggers, ILC3 produce several cytokines, including IL-22, IL-17A/F, GM-CSF and IL-2.

IL-22 maintains barrier integrity through stimulation of epithelial cells turnover (60, 61), induction of tight junction proteins production, anti-bacterial peptides and mucins (62, 63). Vitamins A or D are potent inducers of IL-22 production by murine ILC3 (57, 58), while human ILC3 produce IL-22 after microbial stimulation of phagocytes (64). AhR activation is mandatory for IL-22 expression in mouse ILC3 due to its protein-protein interaction with RORγt (59). For example, L-kynurenine (produced by gut epithelial cells) after ligation to AhR stimulates the proliferation of IL-22^+^ ILC3 (65). Another stimulus for IL-22 production is the activation of G-protein-coupled receptors FFAR on murine ILC3 by the action of SCFA (66, 67). The signaling cues that come from FFAR2 can indirectly affect IL-22 through augmenting expression of the IL-1 receptor and ILC3 responsiveness to IL-1β (66). What is more, IL-23 produced by myeloid cells as a part of an anti-microbial response has the same effect on ILC3 (68).

ILC3-mediated production of IL-17A/F is important for the induction of antimicrobial peptides and tight junction proteins in epithelial cells (69). However, data obtained from both human and murine studies imply that the major role of ILC3-derived IL-17 is to attract neutrophils to the intestinal tissue in response to bacterial (*Mycobacterium tuberculosis* and *Clostridium difficile*) and fungal infections (70–72).

Secretion of GM-CSF and IL-2 from ILC3 is triggered by IL-1β from intestinal macrophages. Mouse ILC3-derived GM-CSF was shown to act upon intestinal macrophages and dendritic cells to promote their production of IL-10 and retinoic acid, that in turn stimulate the induction and enable maintenance of Treg (73). However, ILC3 in the intestine of inflammatory bowel disease patients produce large amounts of GM-CSF that causes a loss in ILC3 and exacerbation of the disease (74). Recently, a very interesting finding was published identifying a population of mouse and human ILC3 that produce IL-2 and are involved in the preservation of oral tolerance through stimulation of Treg differentiation (51). Along with cytokine-mediated activity, ILC3 can modulate adaptive immune response through antigen presentation *via* class II MHC. Namely, ILC3 have the ability to present microbial antigens and to limit CD4^+^ cell response by inducing their cell death (75). The reduction in the specific MHCII^+^ ILC3 population in the intestine is associated with Crohn’s disease in pediatric patients (76).

## ILC3 in T1D

The precise contribution of intestinal ILC3 to the onset and progression of T1D has not been investigated, so far. However, there are some data that emphasize ILC3 as important players in shaping GALT environment for T1D initiation or progression. First, decreased frequency of ILC3 was found in the duodenum of T1D patients (77). The human data are in contrast to total ILC3 increase found in small intestine lamina propria of prediabetic NOD mice (10) and in 20 weeks old NOD mice (our unpublished data). So, the second key statement for hypothetical ILC3 relation to T1D pathology comes from the investigation of ILC3 function. Namely, our preliminary data show lower numbers of potentially protective IL-2-producing ILC3 in small intestine lamina propria in 20 weeks old NOD mice with insulitis and in diabetic C57BL/6 mice with streptozotocin-induced T1D. This was accompanied by down-regulation of FoxP3^+^ Treg number and IL-22 and GM-CSF mRNA expression in the intestine suggesting a causal relationship between IL-2^+^ ILC3 and Treg (unpublished results). Higher number of ILC3 and lower of IL-2-producing ILC3 could point to the pro-inflammatory environment in GALT that is related to T1D pathogenesis. The observed ILC3 reduction in human intestinal biopsies from patients with T1D (77) could be associated with ILC3 ability to convert to IFN-γ-producing ILC1 in the inflammatory environment, a process found both in humans and mice (78, 79). That was surely the case in these T1D patients, as the numbers of ILC1 were significantly increased in the intestinal tissue (77).

The close relationship between gut microbiota and proper function of ILC3 within the pancreas in the prevention of T1D development in mice was identified by Miani et al. (41). T1D in NOD mice was found to be associated with reduced numbers of ILC3 in the pancreas and their down-regulated IL-22 production that led to compromised expression of antimicrobial proteins in the pancreas. In the same study, low IL-22-producing ILC3 were found in pancreatic and mesenteric lymph nodes of diabetic NOD mice. Instead of IL-22, they produced rather significant levels of IFN-γ and TNF. All mentioned findings indicate that the transition from prediabetes to diabetes in NOD mice is associated with impaired ILC3 function that could lead to reduced numbers of Treg and imply the protective role of IL-2^+^ and IL-22^+^ ILC3 against T1D. In general, there are many pathological conditions where ILC3 play a role such as inflammatory bowel disease, experimental autoimmune encephalomyelitis, Graves’ and Hashimoto’s thyroiditis (52, 80–82). Still, further investigation will discriminate whether ILC3 reduction precedes or is the result of ongoing inflammation during T1D pathogenesis.

## Perspectives for ILC3 Modulation in T1D

**Figure 1:**
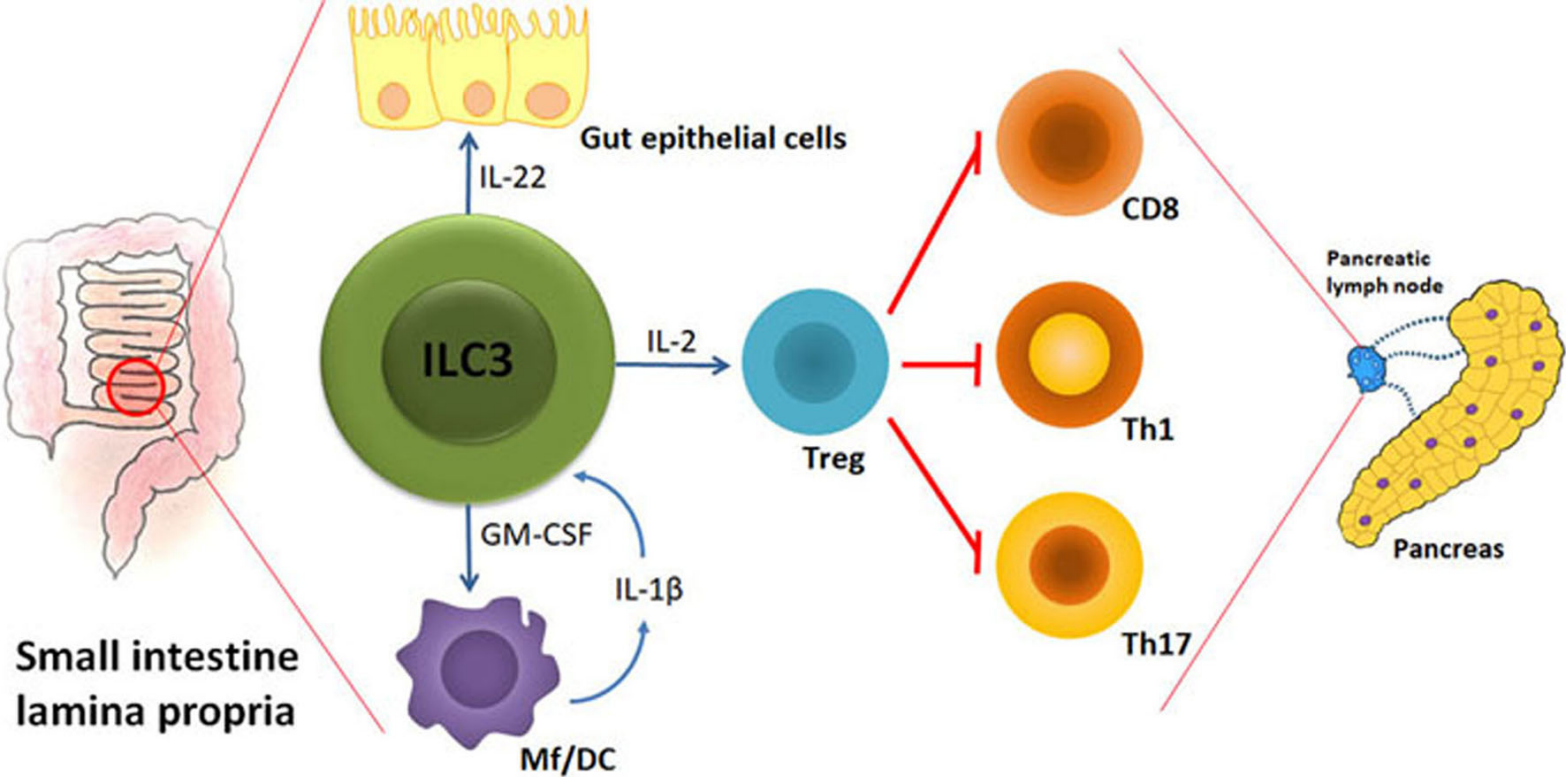
The hypothetical model of ILC3-mediated effects on autoimmune process during T1D. Under the influence of gut microbiota, their metabolites and food ingredients, intestinal ILC3 produce IL-22 that stabilizes the gut barrier and GM-CSF that influences dendritic cells (DC) and macrophages (Mf). Upon activation by microbial cues, Mf produce IL-1β that stimulates ILC3 to increase their production of IL-2 and thus promote intestinal Treg stability and proliferation. Intestinal Treg are able to migrate to the pancreatic lymph nodes and modulate the autoimmune response by providing a suppressive environment in which cytotoxic CD8^+^ cells, Th1 and Th17 cells are inhibited. The final outcome is the blockade of T cell-mediated autoimmune destruction of pancreatic β cells.

There are at least three key ILC3 activities that can counteract initiation and/or progression of T1D: 1. Maintenance of gut barrier integrity; 2. Regulation of gut microbiota homeostasis; 3. Stimulation of Treg proliferation and suppressive function. Therefore, the preserved abundance and function of ILC3 within the intestine could largely aid T1D prevention. The hypothetic model of ILC3 role in protection from autoimmune process during T1D is shown in **Figure 1**.

**Figure 2:**
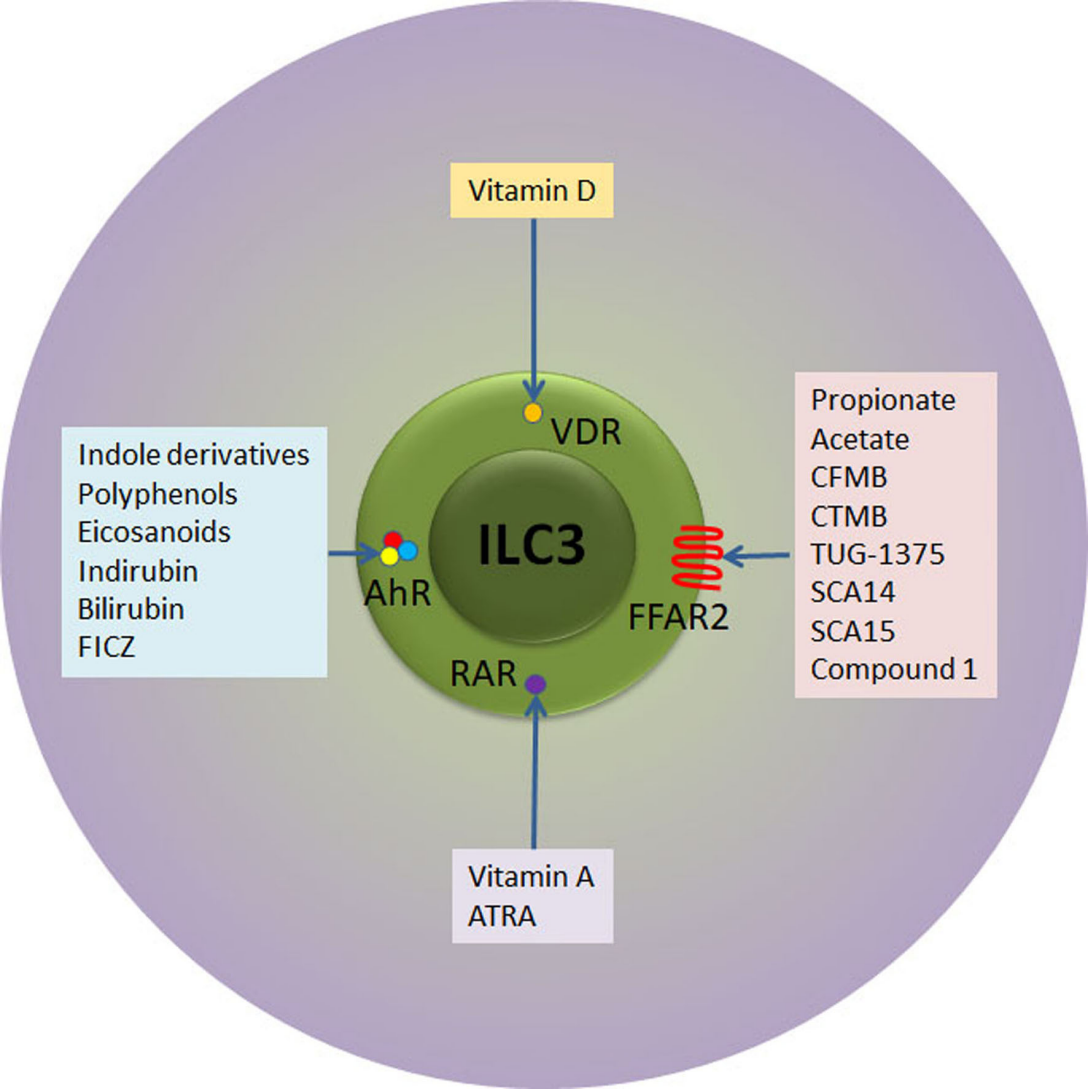
Receptor-ligand interactions relevant for therapeutic targeting of ILC3. ILC3 express receptors for retinoic acid (RAR) and vitamin D (VDR) that upon activation with respective vitamins instigate ILC3 proliferation and/or secretion of IL-22. In addition, ILC3 express AhR transcription factor that can ligate to versatile indol-containing compounds. The activation of AhR is mandatory for the development of mature ILC3 in the intestinal lamina propria, their proliferation and IL-22 secretion. Finally, ILC3 express FFAR2 at very high levels. SCFA (propionate and acetate) as well as several synthetic compounds bind to FFAR2 with high affinity, while Compound 1 and CTMB are selective FFAR2 agonists that promote beneficial ILC3 functions. ATRA, all trans retinoic acid; FICZ, 6-formylindolo[3,2-b]carbazole; CFMB, S)-2-(4-chlorophenyl)-3,3-dimethyl- N-(5-phenylthiazol-2-yl)butamide; 4-CTMB, (S)-2-(4-chlorophenyl)-3- methyl-N-(thiazol-2-yl)butanamide; SCA14, propiolic acid; SCA15, 2-butynoic acid.

As previously stated, there is a number of external stimuli that can be used for ILC3 modulation (**Figure 2**). In addition to stimulation of IL-22 production, vitamin A attracts specifically ILC3 to the intestinal tissue in both mouse and humans (83, 84). Although there are no data about the influence of retinoids on ILC3 during T1D pathogenesis, their effect on Treg stimulation and suppression of pro-inflammatory adaptive and innate immune cells both systemically and within the pancreas was firmly established (85, 86). Indeed, the oral or intraperitoneal application of retinoids showed a significant preventive effect in NOD and streptozotocin-treated C57BL/6 mice (85, 86). Similarly, vitamin D3 (calcitriol) supplementation led to reduced T1D incidence in NOD mice through generation of suppressive environment, including the promotion of Treg (87, 88). Again, similarly to vitamin A, it remains unknown whether the beneficial effect of vitamin D can be attributed to the modulation of ILC3.

Another way of intestinal ILC3 modulation is the application of AhR ligands. The examples of endogenous AhR ligands are eicosanoids, indirubin, bilirubin, or 6-formylindolo[3,2-b]carbazole (89), while exogenous ligands are mainly derived from cruciferous plants (indole-3-carbinol derivatives) (**Figure 2**). In addition to IL-22 stimulation, AhR ligands promote ILC3 survival and proliferation through Notch-dependent pathways (56, 59). The presence of AhR is mandatory for the development of ILC3 in the intestine as AhR-deficient mice show reduced numbers intestinal ILC3, resulting in increased susceptibility to *Citrobacter rodentium* infection (56, 59). Several studies show that AhR activation can prevent T1D and they point to either Treg-dependent mechanisms (90) or Treg-independent mechanisms (91). Again, the role of ILC3 in AhR-mediated protection from T1D remains unknown.

Finally, SCFA can be potent stimulators of ILC3 function. Acetate, propionate and butyrate, gut microbiota metabolites that are released during the digestion of fibers, bind to FFAR2 and FFAR3 expressed on ILC3 surface. FFAR2 is predominantly expressed on intestinal ILC3, compared to other ILC in the gut (55). FFAR2, unlike FFAR3 exerts higher affinity for acetate and propionate, than for butyrate (92).

To date, there are numerous studies that explored the role of SCFA in the prevention of T1D. Oral intake of fibers or purified SCFA decreased disease severity in animal models of T1D. This specialized diet even prevented T1D initiation in the offspring of treated female NOD mice (31, 32, 93–95). In general, the mechanism of SCFA action is mainly attributed to Treg induction. Although considerably effective in animal models, administration of oral butyrate for one month did not affect autoimmune response in individuals with longstanding T1D (27). This effect might be due to the butyrate higher affinity of binding to FFAR3 (92), and its differential effect on different subsets of ILC3 (96). Specifically, butyrate stimulates NKp46^-^ ILC3 that, in addition to IL-22, produce pro-inflammatory cytokines IFN-γ and IL-17 (96).

The fact that FFAR2 is predominantly and highly expressed in the small intestine and colon ILC3 (55) suggests that FFAR2 is the most fitted target for the specific modulation of ILC3. As the highest FFAR2 expression was detected in CCR6^+^ ILC3 subset that predominantly produces IL-22 in response to SCFA (52), the application of FFAR2 ligands implicate even more stringent control of ILC3-mediated immune response within the GALT. The importance of stimulation of ILC3 for autoimmunity prevention or treatment resides in their FFAR2-mediated IL-22 production and proliferation, but also in the fact that this FFAR2-mediated stimulation will not initiate IFN-γ production (97). In addition to natural ligands, several synthetic FFAR2 agonists have been identified so far: class of phenylacetamides that include (S)-2-(4-chlorophenyl)-3,3-dimethyl- N-(5-phenylthiazol-2-yl)butamide (CFMB) and (S)-2-(4-chlorophenyl)-3- methyl-N-(thiazol-2-yl)butanamide (4-CTMB), TUG-1375, propiolic acid (SCA14), 2-butynoic acid (SCA15) and Compound 1 (patent no. WO 2011/076732 A1) (98) (**Figure 2**).

Application of agonists that preferentially bind FFAR2 (such as Compound 1 and 4-CTMB) would increase the probability of beneficial ILC3 activation (52). In contrast to SCFA that activate FFAR2 in such a manner that it couples to either G_i/o_ or G_q_ proteins, Compound 1-activated FFAR2 on ILC3 binds to both proteins (52). The consequence of such FFAR2 activity is increased AKT and STAT3 phosphorylation that lead to up-regulated IL-22 expression in mouse colonic ILC3 (52). FFAR2 agonists may expand their anti-inflammatory effects by binding to FFAR2 expressed on colonic epithelial cells. Specifically, SCFA administration alleviates colonic inflammation in mice by augmenting inflammasome activation in colon epithelial cells (99). However, FFAR2 is relatively highly expressed on mouse pancreatic β cells where it controls (inhibits) glucose-stimulated insulin secretion (100) implicating the use of selective ILC3 stimulators.

Engagement of two different types of receptors on ILC3 might provide even better output, as for example, signals through AhR and FFAR2 integrate at the level of IL-22 expression (69). Another benefit of this joint treatment may be synergistic activation of Treg as they express AhR and FFAR2 as well (52). The consumption of functional foods that contain vitamins A and D, AhR and FFAR ligands may provide the beneficial activation of ILC3. In addition, some of the synthetic compounds, Compound 1 for example, exert rather selective effects on intestinal ILC3 when applied orally (52). The perspective of such compounds is immense as they can control complex cellular interaction within GALT and intestinal barrier and consolidate the anti-inflammatory environment that can lead to prevention or blockade of autoimmunity in pancreas, as well as at other distant sites.

## Data Availability Statement

The original contributions presented in the study are included in the article/supplementary material. Further inquiries can be directed to the corresponding author.

## Author Contributions

IS conceptualized the paper. IS, TS and NP drafted the manuscript. DM revised the manuscript and made Figures. All authors contributed to the article and approved the submitted version.

## Funding

Supported by the Ministry of Education, Science and Technological Development of the Republic of Serbia (451-03-9/2021-14/200007).

## Conflict of Interest

The authors declare that the research was conducted in the absence of any commercial or financial relationships that could be construed as a potential conflict of interest.
